# Fine Structure of the Male Reproductive System and Reproductive Behavior of *Lutzomyia longipalpis* Sandflies (Diptera: Psychodidae: Phlebotominae)

**DOI:** 10.1371/journal.pone.0074898

**Published:** 2013-09-13

**Authors:** Carolina N. Spiegel, Jorge A. C. Bretas, Alexandre A. Peixoto, Felipe M. Vigoder, Rafaela V. Bruno, Maurilio J. Soares

**Affiliations:** 1 Laboratório de Biologia Molecular de Insetos, Instituto Oswaldo Cruz, FIOCRUZ, Rio de Janeiro, RJ, Brazil; 2 Departamento de Biologia Celular e Molecular, Instituto de Biologia, Universidade Federal Fluminense, Outeiro de São João Batista s/n, Valonguinho, Centro, Niterói, RJ, Brazil; 3 Instituto Nacional de Ciência e Tecnologia em Entomologia Molecular – CNPq, Rio de Janeiro, Brazil; 4 Instituto Carlos Chagas, FIOCRUZ, Rua Prof. Algacyr Munhoz Mader, Curitiba, PR, Brazil; University of Melbourne, Australia

## Abstract

**Background:**

The male reproductive system of insects can have several tissues responsible for the secretion of seminal fluid proteins (SFPs), such as testes, accessory glands, seminal vesicles, ejaculatory duct and ejaculatory bulb. The SFPs are transferred during mating and can induce several physiological and behavioral changes in females, such as increase in oviposition and decrease in sexual receptivity after copulation. The phlebotomine *Lutzomyia longipalpis* is the main vector of visceral leishmaniasis. Despite its medical importance, little is known about its reproductive biology. Here we present morphological aspects of the male *L. longipalpis* reproductive system by light, scanning and transmission electron microscopy, and compare the mating frequency of both virgin and previously mated females.

**Results:**

The male *L. longipalpis* reproductive system is comprised by a pair of oval-shaped testes linked to a seminal vesicle by *vasa deferentia*. It follows an ejaculatory duct with an ejaculatory pump (a large bulb enveloped by muscles and associated to tracheas). The terminal endings of the *vasa deferentia* are inserted into the seminal vesicle by invaginations of the seminal vesicle wall, which is composed by a single layer of gland cells, with well-developed endoplasmic reticulum profiles and secretion granules. Our data suggest that the seminal vesicle acts both as a spermatozoa reservoir and as an accessory gland. Mating experiments support this hypothesis, revealing a decrease in mating frequency after copulation that indicates the effect of putative SFPs.

**Conclusion:**

Ultrastructural features of the *L. longipalpis* male seminal vesicle indicated its possible role as an accessory gland. Behavioral observations revealed a reduction in mating frequency of copulated females. Together with transcriptome analyses from male sandfly reproductive organs identifying ESTs encoding orthologs of SFPs, these data indicate the presence of putative *L. longipalpis* SFPs reducing sexual mating frequency of copulated females.

## Background

The sandfly Lutzomyia longipalpis (Lutz & Neiva, 1912) is a species complex [1] and is considered the main vector of visceral leishmaniasis in the New World [2]. This blood-sucking insect is a fascinating model for studies on reproductive behavior as its males form leks on or near hosts [3], releasing sex/aggregation pheromones [4], [5] and producing acoustic signals during copulation [4], [6] that probably play a role in the sexual isolation of different sibling species, as the different species produce diverse combinations of them [1]. The variations observed also suggest a probable differentiation of other aspects of the mating system, as characteristics under sexual selection tend to evolve fast between close related species [7]. In most insects the male reproductive system is formed by one pair of testes linked to a seminal vesicle by vasa deferentia, accessory glands and an ejaculatory duct. However, modifications and adaptations in the male reproductive systems can be found [8]. Studies on the morphology and ultrastructure of internal reproductive organs and spermatozoa in insects offer an important tool in phylogenetic analysis at different taxonomic levels [9], [10].

The testis in Diptera comprises a simple follicle and the follicle wall is a thin epithelium, sometimes consisting of two layers of cells with a basal lamina [8]. Four regions usually constitute the testis follicle: germarium, zone of growth, zone of maturation and zone of transformation, where the sperm develops in successive stages of maturation in the process of spermiogenesis [11], [12]. In some insects the process of spermatogenesis can be completed before adult emergence. This happens mainly in species that do not feed when adult, although the spermatogenesis can continue throughout adult life [8], [9]. In *Drosophila*, the first signals of spermatogenesis are observed as early as in the first instar larval stage [13]. Moreover, the release of mature sperm from the testis into seminal ducts in some insects exhibits a circadian rhythm [14].

Seminal vesicles, where sperm is stored and nourished before transfer to a female, can be originated from different structures according to the insect group analyzed. They consist of dilations of the *vasa deferentia* in many insects [12] or dilations of the ejaculatory duct in some Hymenoptera and Nematoceran Diptera [8].

Many male insects possess glands associated with the reproductive system. These accessory glands synthesize proteins, including the accessory gland proteins, which are transferred to females with sperm during mating, altering their physiology and behavior [15]–[17]. These proteins present a variety of functions that collectively serve to improve male reproductive success. They can also be produced in other secretory tissue from the male reproductive tract, such as testes, seminal vesicles, ejaculatory duct and ejaculatory bulb and therefore they are called in general insect seminal fluid proteins (SFPs) [17]. The SFPs act at all phases of the reproductive biology of the mated female and display different functions, including (a) prevention of subsequent female copula by mating plugs formation, (b) alterations in female behavior such as decreasing receptivity to re-mating and/or reduction in attractiveness, (c) improvement in sperm protection, storage, activation and competition parameters, and (d) induction of changes in female physiology that results in increased oogenesis and oviposition [15]–[17]. SFPs have been characterized in several insect vectors of infectious diseases and can be involved in the reproductive isolation between species [17]–[19].

One of the best functionally characterized SFPs, the sex peptide of *Drosophila* acts as a global regulator of reproductive processes, inducing significant alterations to genes linked to egg development, early embryogenesis, immunity, nutrient sensing and behavior, such as the decreased receptivity of mated females [20]. Correlation between the reduction in re-mating and SFPs has been shown in many different insects, especially in Diptera, and this can be severe to the point where a female only mate once like in some mosquitoes [17], [18], [21]. The decrease in re-mating for a period of time can happen because of female refractoriness (active rejection) [16] and also because of a decline in female atractiveness, making males less likely to court, as observed in *Drosophila* and some Lepidoptera [15], [17], [22].

Although sandflies lack a proper accessory gland, a “plug-like formation” has been observed in the female spermatheca and probably prevents re-insemination [23]. Transcriptome analysis of *L. longipalpis* male reproductive organs has identified 14 ESTs encoding putative SFPs similar to those of *Aedes aegypti*, *Anopheles gambiae* and *D. melanogaster* [24]. Therefore, probably the role of the accessory gland in sandflies is played by other organs such as the seminal vesicle, as suggested by Fausto et al. [25].

Here we have analyzed morphological aspects of the internal reproductive system of male *L. longipalpis* sandflies by light and electron microscopy. In order to obtain an evidence of putative SFPs acting to reduce the mating frequency of mated females, we have also performed behavioral studies.

## Methods

### Insects

*L. longipalpis* sandflies were obtained from a laboratory colony established from insects collected at the Lapinha cave (Minas Gerais State, Brazil, 19°03′S - 43°53′W). The insects were reared at 25°C and 80% ±10% relative humidity (RH), as previously described [26].

### Scanning Electron Microscopy (SEM)

Male adults of 0–6 h after emergence were sedated for 2 minutes in dried CO_2_ atmosphere, dissected and the reproductive organs were fixed by immersion for at least 2 hours in 2.5% glutaraldehyde diluted in 0.1 M sodium cacodylate buffer (pH 7.2). After fixation the samples were washed in 0.1 M sodium cacodylate buffer, post-fixed for 30 minutes in 1% OsO_4_ diluted in 0.1 M sodium cacodylate buffer, washed in this same buffer for 10 minutes, dehydrated in graded acetone series, critical-point dried, mounted with adhesive tape to SEM stubs and coated with a 20-nm gold layer. The material was then observed in a scanning electron microscope.

### Light (LM) and Transmission Electron (TEM) Microscopy

Male insects were sedated for 5 minutes at –20°C and then fixed by immersion in 2.5% glutaraldehyde diluted in 0.1 M sodium cacodylate buffer (pH 7.2). To allow a better tissue fixation, the abdomens were dissected immediately after fixation. The specimens were fixed for 2 hours in 2.5% glutaraldehyde diluted in 0.1 M sodium cacodylate buffer, rinsed twice in this buffer and post-fixed for 1 hour in 1% osmium tetroxide/0.8% potassium ferricyanide/5 mM calcium chloride, diluted in 0.1 M sodium cacodylate buffer. Thereafter, they were rinsed in buffer, dehydrated in ascending acetone series (30%, 50%, 70%, 90%, 2×100%, 10 minutes each) and embedded for 48 hours at 60°C in PolyBed 812 resin.

For TEM, ultra-thin sections were collected on cupper grids, stained with uranyl acetate and lead citrate and then observed by transmission electron microscopy. For LM, semi-thin sections were collected on glass slides and then stained with toluidine blue (for 3 minutes), basic fuchsin (30 seconds) or methylene blue (for 2 minutes). Thereafter the samples were observed in a Nikon Eclipse E600 light microscope. For the LM clarification procedure, insects were immersed for two hours in 10% potassium hydroxide, 20 minutes in acetic acid, 20 minutes in distilled water and finally four hours in lactophenol. Thereafter, the insects were mounted with Berlese liquid and observed in a Nikon Eclipse E600 light microscope.

### Behavioral Analyzes

*L. longipalpis* pupae were separated in micro-centrifuge tubes. After emerging, adults of the same sex were put together in a vial containing small pieces of cotton embedded with sugar solution until the moment of the experiments. Adult couples (2-to-4-day-old females and 4-to-8-day-old males) were observed in a copulating arena illuminated with fluorescent light until the occurrence of copula for a period of at least 20 minutes. The arena consisted of a closed polyethylene Petri dish (0.7 cm in height, 3.5 cm in diameter) with a lateral orifice to introduce the insects. Males age was chosen according to Jones et al. (2000) [27], who showed that when presented with a choice between young (0–2 days after emergence), middle-aged (4–6 day-old adult) and old males (8–10 day-old), females consistently preferred to mate with middle-aged males.

The software EthoLog 2.2.5 [28] was used to register the insect behavior, observing the following parameters: occurrence of copula, time of latency until copula and duration of copula. Mated females were exposed to a second male 24 hours after the first copula, to evaluate a possible reduction in mating frequency.

The occurrence of copula in *L. longipalpis* for virgin (1° copula) and mated (2° copula) females was analyzed by *X^2^* test. Two-tailed t test was performed to evaluate possible differences in time spent to start the first and second copulas, as well as in their duration. Differences were considered significant only when p<0.01.

## Results and Discussion

**Figure 1:**
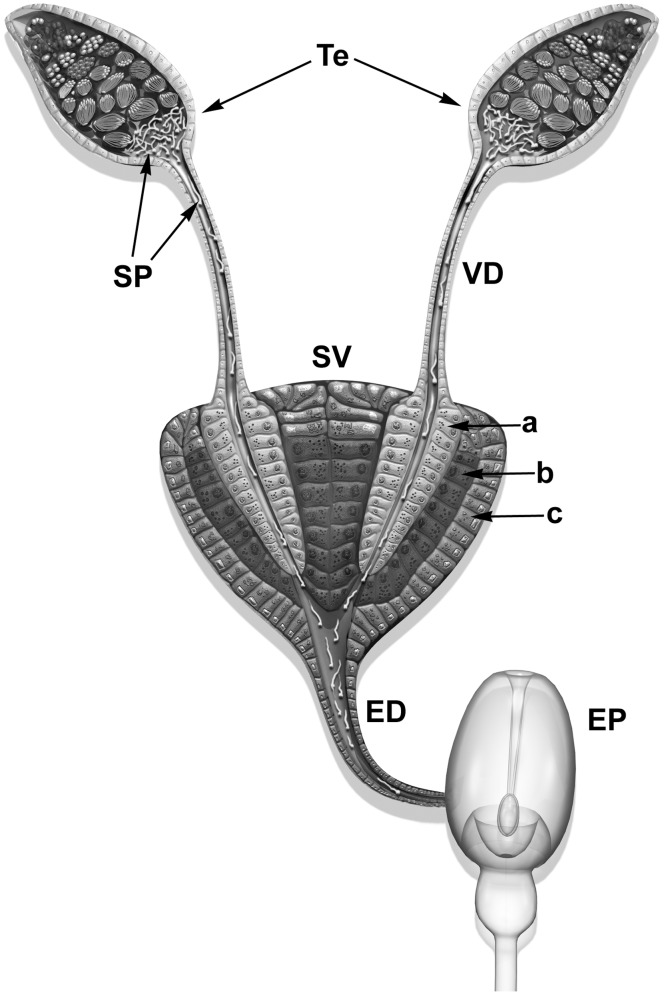
Schematic view of the male reproductive system of *Lutzomia longipalpis*. A pair of testes (Te) is linked via *vasa deferentia* (VD) to a pear-shaped seminal vesicle (SV). An ejaculatory duct (ED) then connects the seminal vesicle to the ejaculatory pump (EP). The *vasa deferentia* are inserted (a) into invaginations (b) of the seminal vesicle wall (c). Cells at layers “a”, “b” and “c” present different types of cytoplasmic granules; SP: mature spermatozoa. Modified from Valdez (2001) and Fausto et al. 2000.

**Figure 2:**
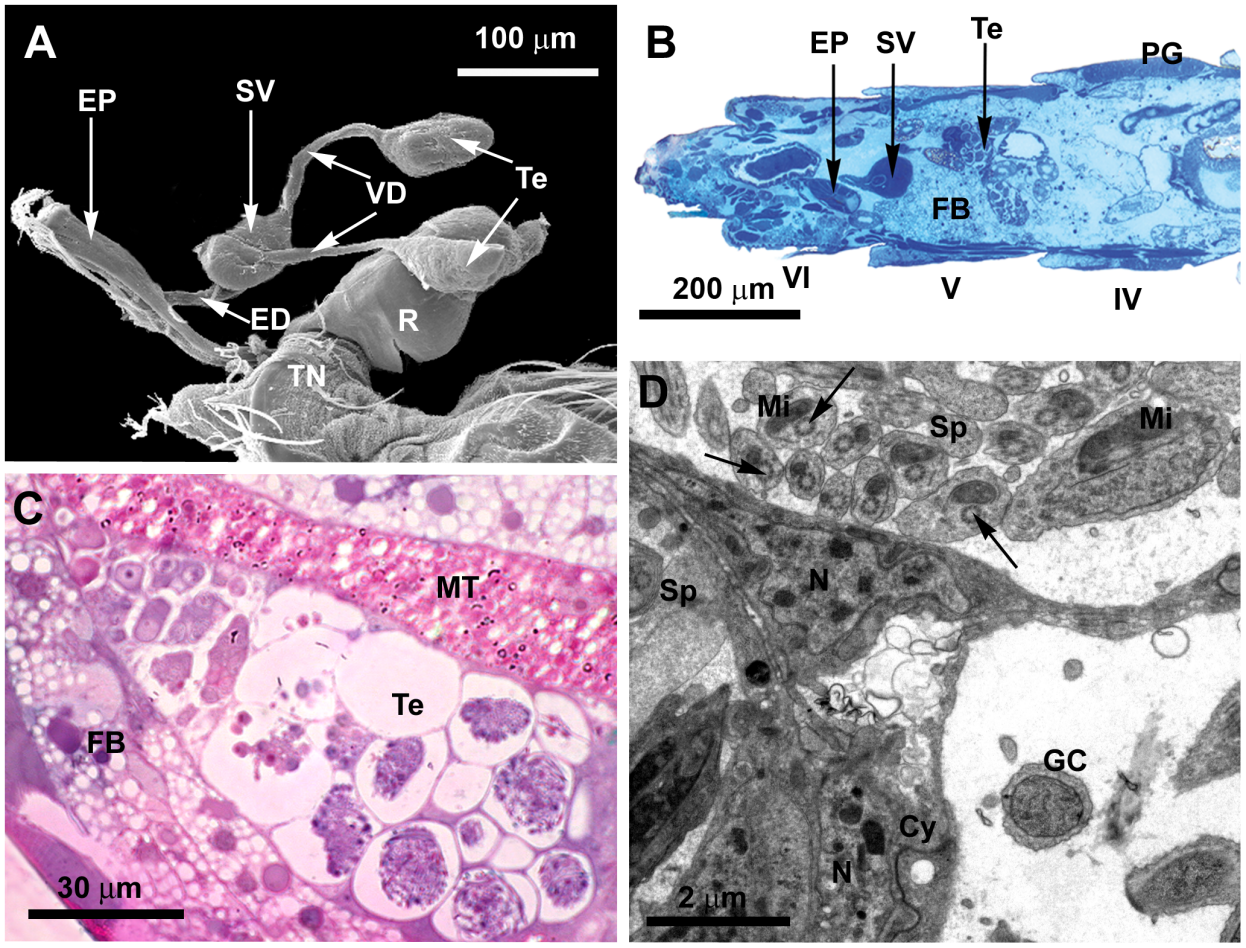
General aspect of the *L. longipalpis* male reproductive system and the testes. **A)** SEM of an isolated male reproductive system, showing the pair of testes (Te), the *vasa deferentia* (VD), the seminal vesicle (SV), the ejaculatory duct (ED), the ejaculatory pump (EP) and the external genitalia or terminalia (TN). Also note the rectum (R). **B)** Light microscopy (LM) section showing the localization of the reproductive organs in the abdomen. Testes (Te) and the seminal vesicle (SV) are located in the fifth abdominal segment (V). Note pheromone gland cells (PG) in the fourth tergite (IV), and the ejaculatory pump (EP) in the sixth abdominal segment (VI). Note the well-developed fat body (FB) surrounding the testis and the seminal vesicle. **C)** LM section of a testis (Te) showing the organization of germ cells with different stages of development along the testis. Note the proximity of Malpighi tubules (MT) and the fat body (FB). **D)** Transmission electron microscopy (TEM) of germ cells (GC) and spermatozoa (Sp) surrounded by cystocytes (Cy), forming the cyst. In the spermatozoa, note the axoneme (arrow) lacking the central pair of microtubules and the mitochondria (Mi) close to it; N: nucleus of the cystocytes.

The reproductive system of *L. longipalpis* males is formed by a pair of oval-shaped testes, each one measuring about 80 µm long×40 µm wide. The testes are linked via a *vas deferens* to a single pear-shaped seminal vesicle, which is then linked to the ejaculatory pump (Fig. 1; Figs. 2A–B). The reproductive system occupies mostly the V-VI abdominal tergites and is surrounded by a well-developed fat body, especially around the testes (Fig. 2B), thus indicating an important participation of the fat body in the reproductive physiology. Indeed, it has been shown that the fat body may produce Takeout, a protein known to influence mating in *Drosophila* [29].

In Diptera the testis consists of a simple sac, which may be considered as a single follicle [8], [11], [12]. The testis of *L. longipalpis* is similar, consisting of a single follicle with germ cells at different stages of development. The pair of testes is located usually at the fifth abdominal segment (Fig. 2B). Germ cells with large nuclei are located at the testes apical portion and can be assembled in groups (Fig. 2C). Clusters of mature spermatozoa are found close to the testes basal portion (Fig. 2D). Each testis, as well as the germ cell assemblies, is enveloped by interdigitated cystocytes with large nuclei and well developed endoplasmic reticulum (Fig. 2D).

**Figure 3:**
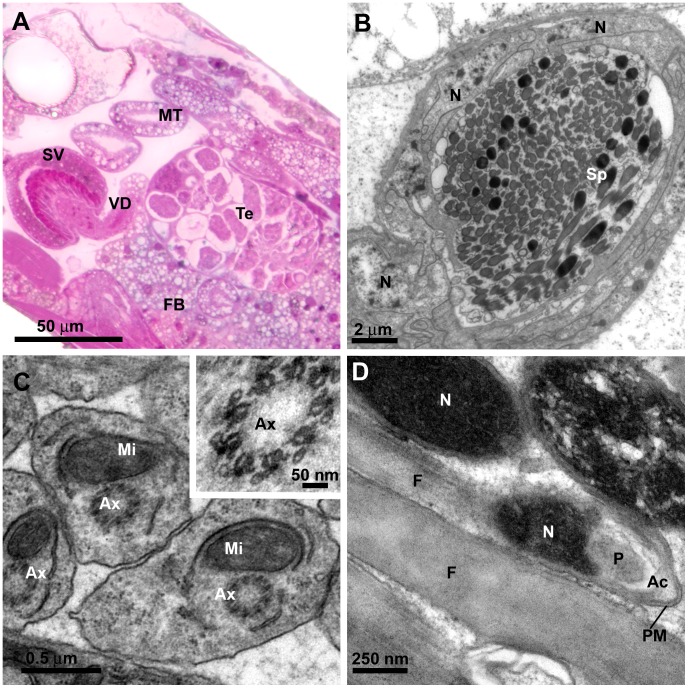
*L. longipalpis* male reproductive system: *vas deferens* and spermatozoa. **A)** LM section of the fifth abdominal segment, showing the testis (Te) and the *vas deferens* (VD) that conducts the sperm from the testis to the seminal vesicle (SV). Note the germ cells inside the testis with different shapes. A well-developed fat body (FB) surrounds the testis and the seminal vesicle; MT: Malpighian tubule. **B)** TEM of the tubular *vas deferens* packed with mature spermatozoa (Sp). It is enveloped by a thin basal membrane and a layer of interdigitated epithelial cells; N: nucleus of the epithelial cell. **C)** Detail of spermatozoa by TEM showing the flagelar axoneme (Ax), which presents a 9+0 array of microtubules; Mi: mitochondrion. A higher magnification of the axoneme is also shown in the inset. **D)** Longitudinal section by TEM of spermatozoa showing flagellum (F), capping perforatorium (P) and the thin acrosomal complex (Ac); PM: plasma membrane; N: meshwork of compact DNA at the spermatozoon head.

The two *vasa deferentia* are inserted into the seminal vesicle through invaginations of the seminal vesicle apical membrane (Fig. 3A). The tubular *vas deferens* is packed with mature spermatozoa (Fig. 3B), enveloped by a thin basal membrane and a layer of interdigitated epithelial cells (Fig. 3B). Spermatozoa were found inside the tubular *vasa deferentia* six hours after adult emergence (Fig. S1). The presence of mature spermatozoa in the *vasa deferentia* was surprising, as males of this species are sexually mature only 24 hours after emergence, when the external genitalia has completed its rotation [30]. However, it is known that short-lived insect males usually present mature spermatozoa after emergence and the whole process of spermatogenesis may be complete before adult eclosion [8].

The axoneme of flagellated spermatozoa presented a 9+0 array of microtubules (Fig. 3C, inset). The spermatozoon head is occupied by a meshwork of compact DNA, with an acrosomal complex at its anterior end comprising a vesicular acrosome and a perforatorium (Fig. 3D). Studies of sperm morphology and ultrastructure are important in species phylogenesis, with some sperm structural characters highly conserved and others extremely divergent [9]. The flagellum of this New World sandfly is very similar to those of other previously described phlebotomines *Phlebotomus papatasii* [31], *P. perfiliewi* and *P. perniciosus* [32].

**Figure 4:**
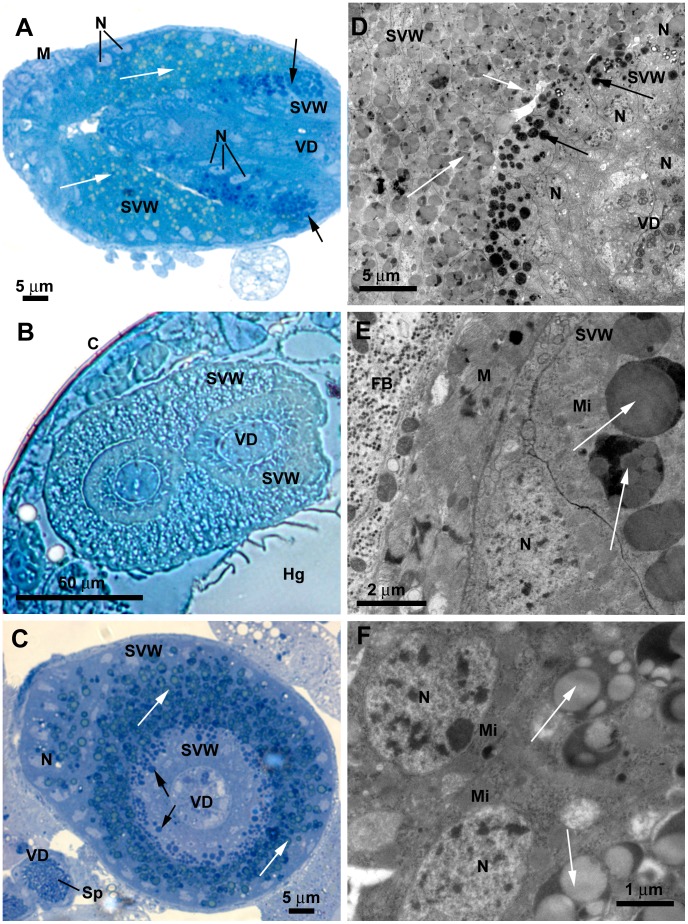
*L. longipalpis* male reproductive system: seminal vesicle, general aspects and the external epithelium. **A)** LM longitudinal section through the seminal vesicle shows that it is surrounded by muscle cells (M) and formed by three consecutive distinct layers of cells: the outer epithelial seminal vesicle wall (SVW), the denser, invaginated epithelial wall of the seminal vesicle and the inner circular epithelial layer of the *vas deferens* (VD). Note the basal localization of the nuclei (N) and the different granule types inside the cells at the inner and outer seminal vesicle wall (black and white arrows, respectively). **B)** LM transversal section of the seminal vesicle showing the insertion of the terminal endings of the *vasa deferentia* (VD), forming two lobes. Note the folded seminal vesicle wall (SVW) around the *vasa deferentia*; C: Cuticle; Hg: Hindgut. **C)** LM section showing the different spherical granules (arrows) in the cytoplasm of the cells of the seminal vesicle wall (SVW), and the basal location of the nuclei (N); Sp: spermatozoon; VD: *vas deferens*. **D)** TEM showing the cells at the outer seminal vesicle wall (SVW) with several cytoplasmic granules (white arrows) with different electron-density as compared to that of granules in the invaginated seminal vesicle wall (black arrows). Note the basal location of the nuclei (N) and the inner *vas deferens* (VD). **E)** TEM image showing the muscle fibers (M) and the fat body (FB) that envelope the seminal vesicle. The arrows indicate the dense granules; N: nucleus; Mi: mitochondria; SVW seminal vesicle wall. **F)** Detail by TEM showing the large nucleus (N) and mitochondria (Mi) located at the basal portion of cells at the peripheral epithelium of the seminal vesicle wall. The cytoplasmic granules are not membrane bound and appear to contain lipid droplets of different densities (arrows).

In *L. longipalpis* males the seminal vesicle is a single organ where the two *vasa deferentia* are deeply inserted into its lumen, by running inside invaginations of the seminal vesicle single-layered epithelium. Thus, an invaginated seminal vesicle epithelium envelops each *vas deferens*, forming muff-like structures (Fig. 4A). The seminal vesicle measures about 100 µm long x 60 µm wide. Longitudinal sections show that it is contains by three consecutive distinct layers of cells, which correspond to the outer epithelial layer of the seminal vesicle, the invaginated epithelial layer of the seminal vesicle and the circular epithelial layer of the inserted *vas deferens* (Figs. 4A–D).

The seminal vesicle wall is formed by a single layer of large cubic cells. These cells have the nuclei and mitochondria located at the basal portion, while several electron-lucent granules and endoplasmic reticulum profiles are found at the apical portion (Figs. 4E–F). The round nucleus presents dispersed heterochromatin. The cytoplasmic granules are not membrane bound and appear to contain lipid droplets of different densities (Figs. 4E–F). Occasionally the inclusions of these cytoplasmic granules present a crystalloid shape (Fig. S1). The seminal vesicle is enveloped by two orthogonal layers of muscle cells (Fig. 4E) that probably contribute to expel the sperm during mating. Tracheas are observed associated to the seminal vesicle, suggesting a high metabolic activity in this organ, since tracheas may supplement oxygen for the muscular activity (Fig. S1).

**Figure 5:**
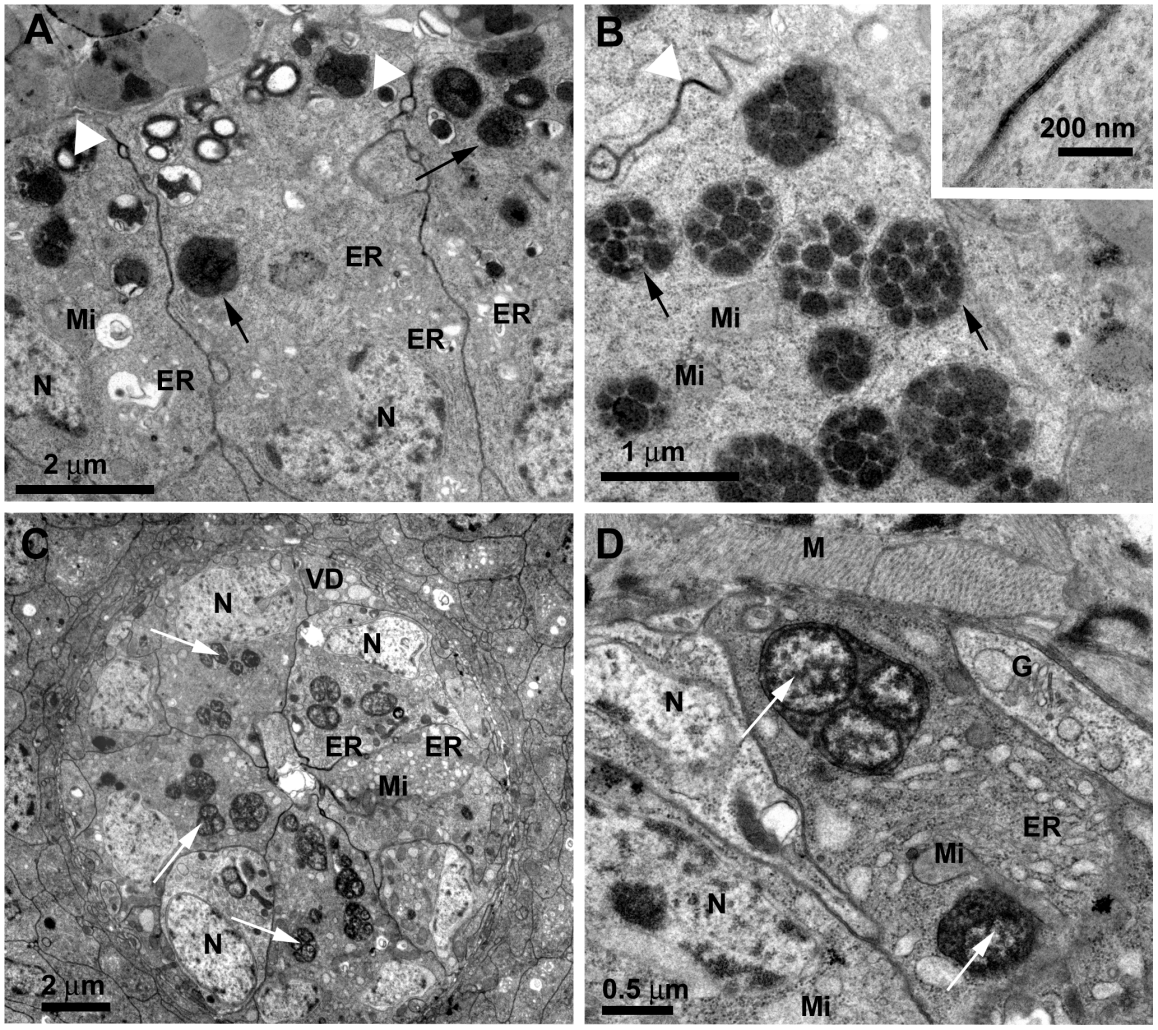
*L. longipalpis* male reproductive system: inner seminal vesicle wall and *vas deferens.* A**)** TEM image showing cells from the inner seminal vesicle wall. They present well-developed endoplasmic reticulum (ER) and the cytoplasmic granules (arrows) are more compact than those of cells from the outer layer; the cells are joined by septate junctions (white arrowhead); N: Nucleus; Mi: Mitochondria. B) Detail by TEM showing the dense granules (arrows) of cells from the inner seminal vesicle wall: they are membrane bound and contain several electron dense vesicles; Mi: mitochondria. A detail of a septate junction (white arrowhead) is shown in the inset. **C)** TEM image showing cells at the inner *vas deferens* (VD). They present well-developed endoplasmic reticulum (ER), cytoplasmic granules (arrows) and a large ovoid nucleus (N) at the basal portion; Mi: Mitochondria. **D)** Detail by TEM of cells at the inner *vas deferens*: They contain mitochondria (Mi), Golgi complex (G), large amount of swollen endoplasmic reticulum cisternae (ER) and present membrane-bound cytoplasmic granules that contain one to several vesicles, each presenting peripheral electron-density (arrows); the *vas deferens* wall is enveloped by a thin layer of muscle cells (M); N: nucleus.

Cells of the invaginated seminal vesicle epithelium are more electron-dense, presenting well-developed endoplasmic reticulum profiles, Golgi complexes, mitochondria and a large number of dense cytoplasmic granules located at the apical portion (Figs. 5A–B). Since this layer represents an infolding of the seminal vesicle epithelium, its apical portion is facing the apical portion of the outer epithelium (Fig. 5A). These cells, likewise all cells in the seminal vesicle, are kept together by septate junctions (Fig. 5B, inset). The dense granules are membrane bound and contain several round electron dense vesicles (Fig. 5B).

The *vas deferens* wall inside the seminal vesicle is formed by small, interdigitating, electron-lucent cells with a large round nucleus, disposed around a central lumen (Fig. 5C). These cells present mitochondria, Golgi complex, large amount of swollen endoplasmic reticulum cisternae and membrane-bound cytoplasmic granules that contain one to several vesicles, each presenting peripheral electron-density (Figs. 5C–D). The *vas deferens* wall is enveloped by a thin layer of muscle cells (Fig. 5D).

The seminal vesicle serves as a storage reservoir for sperm before it is transferred to the female [8]. In *L. longipalpis* sandflies the lumen of the seminal vesicle probably works for sperm storage, whereas the cells of the seminal vesicle around the inserted *vasa deferentia* possibly produce and secrete specific products, such as proteins and peptides, as in the accessory glands of other insects. The presence of dense cytoplasmic granules and well-developed endoplasmic reticulum profiles in cells at the seminal vesicle wall is a typical feature of gland cells.

**Figure 6:**
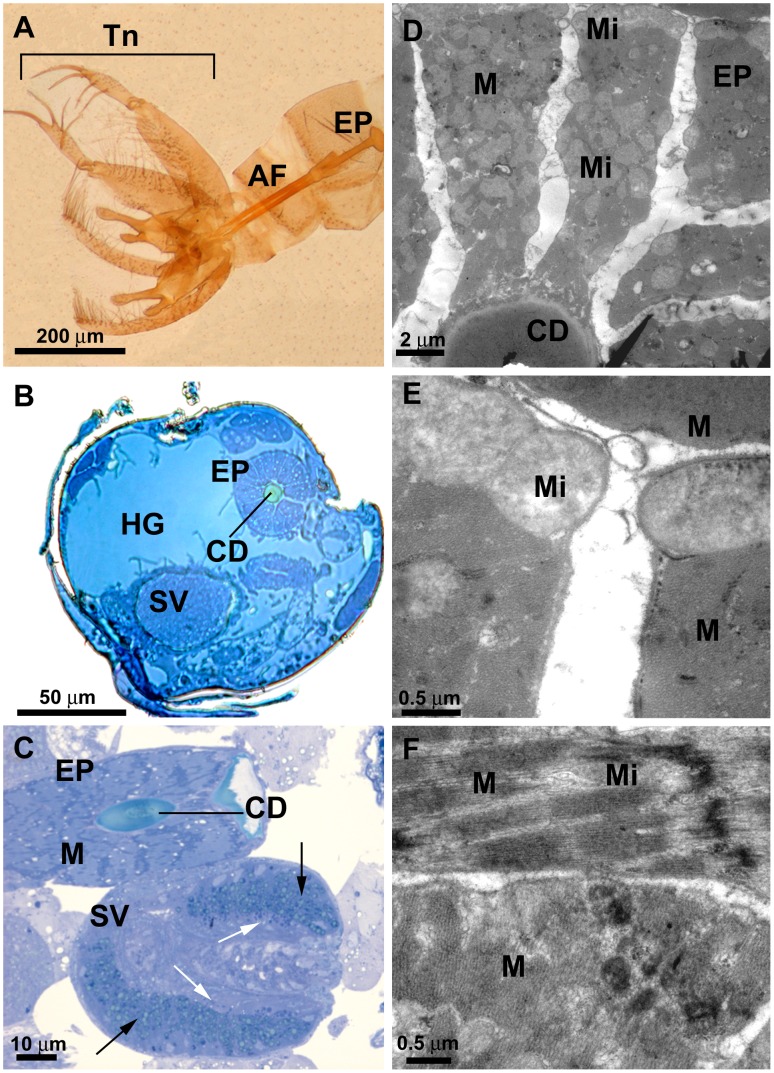
*L. longipalpis* male reproductive system: the ejaculatory pump. **A)** LM detail of the cuticular ejaculatory pump (EP) and the two aedeagal filaments (AF). Note the external genitalia, or terminalia (Tn), formed by modification of the eighth, ninth and tenth abdominal segments. **B)** LM transversal section of the ejaculatory pump (EP) showing the central cuticular duct (CD); HG: Hind gut; SV: seminal vesicle. **C)** LM longitudinal section of the ejaculatory pump (EP) showing well-developed muscle fibers (M) surrounding a central cuticular duct (CD). Below is a longitudinal section through the seminal vesicle (SV) at the point of insertion of a *vas deferens* (to the right), showing the two consecutive seminal vesicle cell layers with different granule types (black and white arrows). **D)** TEM transversal section of the ejaculatory pump (EP) showing the muscle fibers (M) with mitochondria (Mi) surrounding the cuticular duct (CD) **E)** TEM detail of the muscle fibers (M) with mitochondria (Mi). **F)** TEM detail showing the disposition of the muscle fibers (M); The thin outer sheet is disposed transversally. Mi: mitochondria.

A cuticular ejaculatory duct is located after the seminal vesicle and is surrounded by well-developed muscles, forming the ejaculatory pump (Figs. 6A–C). The ejaculatory pump of *L. longipalpis* is similar to that of the Mexican fruit fly (Diptera: Tephrididae) [11], both being formed by a large bulb surrounded by muscles (Figs. 6D–F). The presence of cuticle (Figs. 6A–C) reflects the ectodermic origin of this organ, as previously noted by Foratini [33]. The ejaculatory pump is connected to the copulatory organ, composed by two cutinized aedeagal filaments, thus constituting the penis of this insect. The two aedeagal filaments are located from the 8^th^ abdominal segment to the last segments that form the male terminalia (Fig. 6A). Several tracheas are observed associated to the ejaculatory pump, which reflects the high metabolic activity when contracting to expel the sperm (Fig. S1).

### Reproductive Behavior

The nature and function of substances synthesized by the seminal vesicle of *L. longipalpis* is unknown, but they may contribute to sperm maturation and formation of mating plugs, such as SFPs of other insects. In some insects it has been described that in the absence of accessory glands their role may be overtaken by glandular cells in the seminal vesicles, *vasa deferentia*, or the ejaculatory duct [15], [34].

To verify the effect of putative SFPs, behavioral analyses were performed and showed a clear reduction in the frequency of a second copula after 24 hours (Table 1). While 42% of virgin females were observed to copulate, only 25% females engaged in a second copula 24 h later. This significative reduction in occurrence of copula in already-mated females supports the hypothesis of a possible presence of SFPs changing the sexual behavior of females, as described in other insects [35], [36]. In *Drosophila*, the seminal fluid not only makes females less likely to have a second copulation for a period of time [16], but it also drops female attractiveness [22]. According to Bray and Hamilton [37], male sandflies initiate copulation after successful courtship depending on a chain of signals and responses, encompassing both male and female behaviors. Therefore, further studies are necessary in sandflies to identify if the reduction of mating frequency after the first copula is due to female rejection, reduction of her attractiveness or both.

**Table 1:** Mating frequency of virgin and mated females.

Female	N° of trials	N° of copulas	Copula (%)
Virgin	272	115	42
Mated	115	29	25

Occurrence of copula in *L. longipalpis* for virgin (1° copula) and mated (2° copula) females. A single pair (1 male - 1 female) was used in each trial. X^2^ = 10.071 and p<0.01.

Moreover, from the 25% of females that mated for the second time, there is a possibility that seminal fluid was not transferred during the first copula (incomplete copula), and thus these females did not receive SFPs. This hypothesis is supported by the fact that only 76% of *L. longipalpis* mated females (population of Lapinha) produced eggs before dying [38], possibly due to the absence of seminal fluid and sperm transference. The observation that mating trials between different species of the *L. longipalpis* complex resulted in copulation, but no insemination, indicates that genital contact does not necessarily mean insemination success [3].

Jones [39] found similar results in her study on the cost of monandry in the lekking sandfly. When mated blood-fed females from Salvaterra population (a different sibiling species of the *L. longipalpis* complex) were exposed to a new aggregation of males, they were reluctant to re-mate in a single gonotrophic cycle, irrespective of the time elapsed since their first mating 1, 24, or 72 h after the first copula. Only 18% of females engaged in a second mating attempt.

Blood feeding is an important stimulus for egg production in haematophagous insects [8] and can interfere in female mating receptivity in triatomine bugs [40], [41] or even stimulate the release of sperm from the spermatophore in *Phlebotomus* [31], [42]. Thus, it is interesting to note that in *L. longipalpis* a decrease in mating frequency was found 24 h after the first copula, even with females fed only in sugar solution. Despite its medical importance, little is known about the reproductive biology in the *L. longipalpis* sibling species complex and differences in their life cycle have been reported under laboratory conditions [43]. In *P. duboscqui* it has been suggested that females mate only once per gonotrophic cycle, as the males made no attempt to court or did not display the typical behavior of mating recognition called “piggy-backing” with recently mated females [44]. Furthermore, a ‘plug-like formation’ has been observed in the spermatheca of other sandflies such as *P. perniciosus*, *P. papatasi* [23] and *P. duboscqui* [44] and probably acts to prevent female re-insemination, although there are some studies suggesting that it was a misinterpretation and that females of *P. papatasi* are polyandrous [31].

The low rate of occurrence of the first copula with virgin females (3–5-day-old, fed with sugar only), was similar to that previously obtained by Souza et al. [38] using 5–7-day-old virgin bloodfed females of *L. longipalpis* (Lapinha population) and also by Bray and Hamilton [37] working with a Jacobina population and virgin females fed only in sugar solution. Although all our tested sandflies were virgins of breeding age, less than half of male–female pairs mated during the 20 min trials.

**Figure 7:**
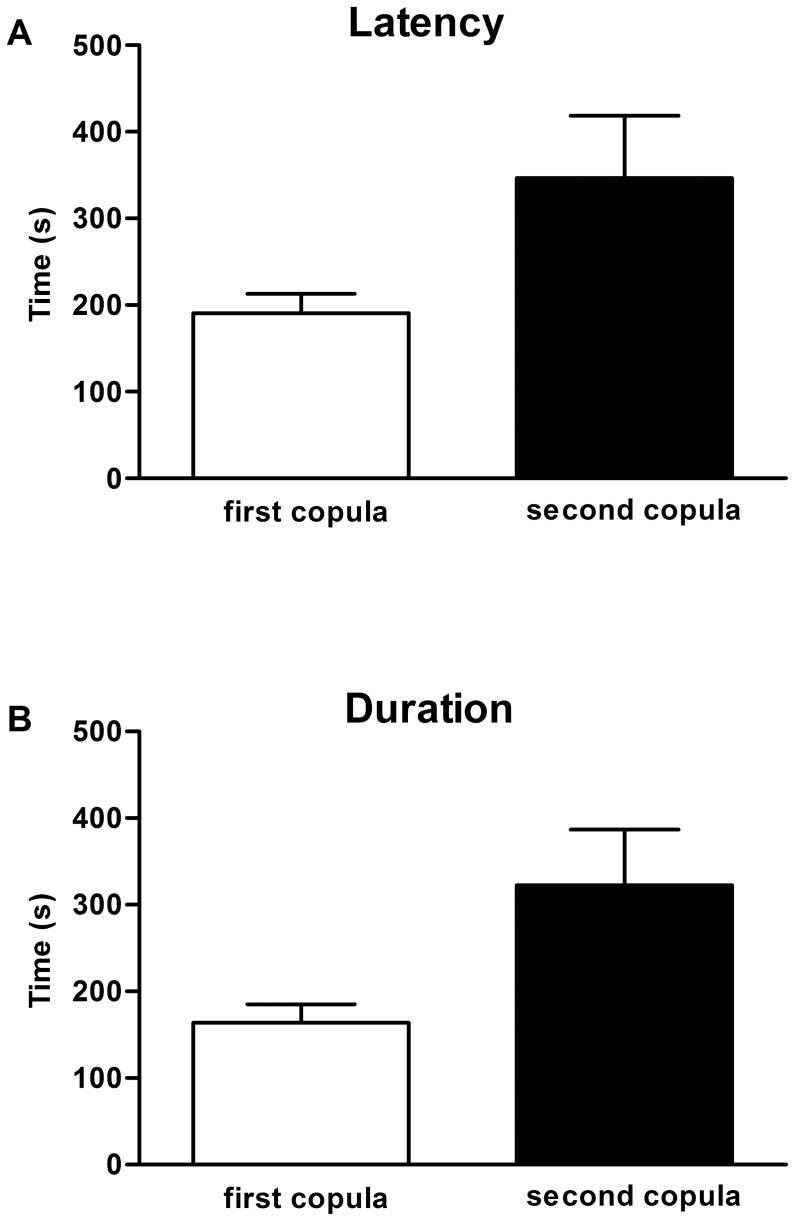
Behavior analysis. **(A)** Latency to start copula in virgin females (first copula) and already-mated females (second copula). Two-tailed t test, p<0.01. **(B)** Duration of copula of virgin females (first copula) and already-mated females (second copula). Two-tailed t test, p<0.01.

Virgin females accepted the males and initiated copula significantly faster than already mated females (Fig. 7A), thus showing a shorter latency period. On average, virgin females initiated the copula within the first 190 seconds, while mated females showed a latency of 346 seconds. This result confirms the idea that either the females are resistant to a second copula or males are less attracted to them. Moreover, duration of the first copula was significantly lower than the second one: on average 164 seconds, versus 322 seconds (Fig. 7B). It is possible that already-mated females, which already received SFPs in the first copula, hinder the correct positioning of the male penial filaments. Thus, the time necessary for a second copula is greater and possibly the male quits before transference of sperm to the female genital tract. Alternatively, already mated females are “choosier” and males have to perform longer copulatory courtships, which as mentioned before involve the production of copulation songs [6], before succeeding in inseminating these females. Varied copulation duration were also observed in Jacobina populations [45], but the relationship between copulation duration and insemination success in *L. longipalpis* remains unknown. In *Drosophila*, mating duration was always significantly longer in matings involving males exposed to rivals prior to mating. It seems that the males exert control over the duration of extended matings in response to the potential level of sperm competition [46]. Therefore, it is also possible that *L. longipalpis* males invest more time in a mated female.

Altogether, the ultrastructural features of the seminal vesicle indicating its possible role as an accessory gland, the behavioral studies revealing a reduction in mating frequency of mated females and the transcriptome analysis of male reproductive organs that identified ESTs encoding orthologs of SFPs from other insects [24], indicate the effect of putative *L. longipalpis* SFPs acting on the reduction of a second copula of mated females. Further studies on the biochemistry, physiology and molecular biology of the seminal vesicle are needed for a better understanding of the role of possible protein secretions in the reproductive process of this sandfly.

## Conclusions

Morphological aspects of the male reproductive system of *L. longipalpis* were here characterized by light, scanning and transmission electron microscopy. It is formed by a pair of testes, a seminal vesicle and an ejaculatory duct constituted by the ejaculatory pump and the penial filaments. The seminal vesicle contains typical gland cells, indicating its possible role as an accessory gland. Mating experiments support this hypothesis, revealing a reduction in mating frequency of mated females that indicates the effect of putative SFPs.

## Supporting Information

Figure S1 *L. longipalpis* male reproductive system. **A)** TEM detail of cytoplasmic granules presenting a crystalloid inclusion (asterisk) in cells at the peripheral epithelium of the seminal vesicle wall. Note the difference in density between granules in cells from the outer epithelial wall (white arrows) and in cells from the invaginated epithelium of the seminal vesicle wall (black arrows). **B)** SEM section of a testis (Te), with groups of germ cells (gc) in primary stage of development. Note the cyst (asterisks) formed by germ cells and cystocytes. The seminal vesicle (sv), a *vas deferens* (vd) and trachea (tc) can be also observed. **C)** SEM section of a *vas deferens* (vd) from an insect six hours after emergence. Note the presence of flagellate spermatozoa (arrows). **D)** SEM detail of the ejaculatory duct, formed by a central bulb, and the ejaculatory bulb (eb) surrounded by compressor muscles (asterisks) associated with several tracheas (tc).
(TIF)

## References

[1] Araki AS, Vigoder FM, Bauzer LGSR, Ferreira GEM, Souza NA, et al. (2009) Molecular and behavioral differentiation among Brazilian populations of *Lutzomyia longipalpis* (Diptera: Psychodidae: Phlebotominae). PLoS Neglect Trop Dis 3: e365. Available: http://www.pubmedcentral.nih.gov/articlerender.fcgi?artid=2628317&tool=pmcentrez&rendertype=abstract. Accessed 2013 Aug 16. 10.1371/journal.pntd.0000365 PMC2628317 19172187

[2] Lainson R, Rangel EF (2005) *Lutzomyia longipalpis* and the eco-epidemiology of American visceral leishmaniasis, with particular reference to Brazil: a review. Mem Inst Oswaldo Cruz 100: 811–827. Available: http://www.bioline.org.br/pdf?oc05169. Accessed 2013 Aug 16. 10.1590/s0074-02762005000800001 16444411

[3] Kelly DW, Dye C (1997) Pheromones, kairomones and the aggregation dynamics of the sandfly *Lutzomyia longipalpis* Anim Behav 53: 721–731. Available: http://www.sciencedirect.com/science/article/B6W9W-45MFNR4-13/2/b70fce000fc5ccde6ad07bef0f83fad7. Accessed 2013 Aug 16.

[4] Ward R, Phillips A, Burnet B, Marcondes C (1988) The *Lutzomyia longipalpis* complex: reprodution and distribution. MW Service, Biosystematics of Haematophagous Insects. Oxford: Oxford University Press. 258–269.

[5] Spiegel CN, Jeanbourquin P, Guerin PM, Hooper AM, Claude S, et al. (2005) (1S,3S,7R)-3-methyl-alpha-himachalene from the male sandfly *Lutzomyia longipalpis* (Diptera: Psychodidae) induces neurophysiological responses and attracts both males and females. J Insect Physiol 51: 1366–1375. Available: http://www.sciencedirect.com/science/article/pii/S0022191005001812. Accessed 2013 Aug 16. 10.1016/j.jinsphys.2005.08.007 16226273

[6] Souza NA, Vigoder FM, Araki AS, Ward RD, Kyriacou CP, et al. (2004) Analysis of the copulatory courtship songs of *Lutzomyia longipalpis* in six populations from Brazil. J Med Entomol 41: 906–913. Available: http://www.bioone.org/doi/pdf/10.1603/0022-2585-41.5.906. Accessed 2013 Aug 16. 10.1603/0022-2585-41.5.906 15535620

[7] Ritchie MG (2007) Sexual Selection and Speciation. Ann Rev Ecol Evol Syst 38: 79–102. Available: http://www.annualreviews.org/doi/abs/10.1146/annurev.ecolsys.38.091206.095733. Accessed 2013 Aug 16.

[8] Chapman RF (1998) The Insects: Structure and Function. 4^th^ ed. New York: Cambridge University Press. 788 p.

[9] Birkhead TR, Hosken DJ, Pitnick S, editors (2009) Sperm Biology: an evolutionary perspective. 1^st^ ed. London: Academic Press, Elsevier. 642 p.

[10] Quicke DLJ, Ingram SN, Baillie HS, Gaitens PV (1992) Sperm structure and ultrastructure in the Hymenoptera (Insecta). Zool Scripta 21: 381–402. Available: http://onlinelibrary.wiley.com/doi/10.1111/j.1463-6409.1992.tb00339.x/abstract. Accessed 2013 Aug 16.

[11] Valdez J (2001) Ultrastructure of the testis of the Mexican fruit fly (Diptera:Tephritidae). Ann Entomol Soc Am 94: 251–256. Available: http://www.bioone.org/doi/pdf/10.1603/0013-8746(2001)094%5B0251%3AUOTTOT%5D2.0.CO%3B2. Accessed 2013 Aug 16.

[12] Dallacqua RP, Da Cruz-Landim C (2003) Ultrastructure of the ducts of the reproductive tract of males of *Melipona bicolor bicolor lepeletier* (Hymenoptera, Apinae, Meliponini). Anat Histol Embryol 32: 276–281. Available: http://www.ncbi.nlm.nih.gov/pubmed/12969027. Accessed 2013 Aug 16. 10.1046/j.1439-0264.2003.00484.x 12969027

[13] Casper A, Van Doren M (2006) The control of sexual identity in the *Drosophila* germline. Development 133: 2783–2791. Available: http://www.ncbi.nlm.nih.gov/pubmed/16835435. Accessed 2013 Aug 16. 10.1242/dev.02415 16835435

[14] Giebultowicz JM, Riemann JG, Raina AK, Ridgway RL (1989) Circadian system controlling release of sperm in the insect testes. Science 245: 1098–1100. Available: http://www.sciencemag.org/content/245/4922/1098.refs. Accessed 2013 Aug 16. 10.1126/science.245.4922.1098 17838810

[15] Gillott C (2003) Male accessory gland secretions: modulators of female reproductive physiology and behavior. Annu Rev Entomol 48: 163–184. Available: http://www.ncbi.nlm.nih.gov/pubmed/12208817. Accessed 2013 Aug 16. 10.1146/annurev.ento.48.091801.112657 12208817

[16] Wolfner MF (1997) Tokens of love: functions and regulation of *Drosophila* male accessory gland products. Insect Biochem Mol Biol 27: 179–192. Available: http://www.sciencedirect.com/science/article/pii/S0965174896000847. Accessed 2013 Aug 16. 10.1016/s0965-1748(96)00084-7 9090115

[17] Avila FW, Sirot LK, LaFlamme BA, Rubinstein CD, Wolfner MF (2011) Insect seminal fluid proteins: identification and function. Annu Rev Entomol 56: 21–40. Available: http://www.ncbi.nlm.nih.gov/pubmed/20868282. Accessed 2013 Aug 16. 10.1146/annurev-ento-120709-144823 PMC3925971 20868282

[18] Baldini F, Gabrieli P, Rogers DW, Catteruccia F (2012) Function and composition of male accessory gland secretions in *Anopheles gambiae*: a comparison with other insect vectors of infectious diseases. Pathogens and global health 106: 82–93. Available: http://www.ncbi.nlm.nih.gov/pubmed/22943543. Accessed 2013 Aug 16. 10.1179/2047773212Y.0000000016 PMC4001493 22943543

[19] Helinski MEH, Deewatthanawong P, Sirot LK, Wolfner MF, Harrington LC (2012) Duration and dose-dependency of female sexual receptivity responses to seminal fluid proteins in *Aedes albopictus* and *Ae. aegypti* mosquitoes. J Insect Physiol. Available: http://www.ncbi.nlm.nih.gov/pubmed/22796224. Accessed 2013 Aug 16. 10.1016/j.jinsphys.2012.07.003 PMC3438290 22796224

[20] Gioti A, Wigby S, Wertheim B, Schuster E, Martinez P, et al. (2012) Sex peptide of Drosophila melanogaster males is a global regulator of reproductive processes in females. Proc R Soc B 279: 4423–4432. Available: http://www.ncbi.nlm.nih.gov/pubmed/22977156. Accessed 2013 Aug 16. 10.1098/rspb.2012.1634 PMC3479807 22977156

[21] Yamane T, Miyatake T, Kimura Y (2008) Female mating receptivity after injection of male-derived extracts in *Callosobruchus maculatus* J Insect Physiol 54: 1522–1527. Available: http://www.sciencedirect.com/science/article/B6T3F-4TDK70Y-1/2/89510363a227e16e0df9619d1f282a74. Accessed 2013 Aug 16. 10.1016/j.jinsphys.2008.09.001 18831977

[22] Tram U, Wolfner MF (1998) Seminal fluid regulation of female sexual attractiveness in *Drosophila melanogaster* Proc Natl Acad Sci USA 95: 4051–4054. Available: hhttp://www.ncbi.nlm.nih.gov/pmc/articles/PMC19961/. Accessed 2013 Aug 16. 10.1073/pnas.95.7.4051 PMC19961 9520491

[23] Maroli M, Bettini S, Tricoli D, Khoury C, Perrotti E (1991) Studies on mating plug of two sandfly species, *Phlebotomus perniciosus* and *Phlebotomus papatasi* (Diptera: Psychodidae). Parassitologia 33 Suppl: 405–411. Available: http://www.ncbi.nlm.nih.gov/pubmed/1841236. Accessed 2013 Aug 16. 1841236

[24] Azevedo RVDM, Dias DBS, Bretas JAC, Mazzoni CJ, Souza NA, et al. (2012) The transcriptome of *Lutzomyia longipalpis* (Diptera: Psychodidae) male reproductive organs. PLoS ONE 7: e34495. Available: http://dx.plos.org/10.1371/journal.pone.0034495. Accessed 2013 Aug 16. 10.1371/journal.pone.0034495 PMC3320635 22496818

[25] Fausto AM, Gambellini G, Taddei AR, Maroli M, Mazzini M (2000) Ultrastructure of the seminal vesicle of *Phlebotomus perniciosus* Newstead (Diptera, Psychodidae). Tissue Cell 32: 228–237. Available: http://www.ncbi.nlm.nih.gov/pubmed/11037793. Accessed 2013 Aug 16. 10.1054/tice.2000.0110 11037793

[26] Modi GB, Tesh RB (1983) A simple technique for mass rearing *Lutzomyia longipalpis* and *Phlebotomus papatasi* (Diptera: Psychodidae) in the laboratory. J Med Entomol 20: 568–569. Available: http://www.ncbi.nlm.nih.gov/pubmed/6644754. Accessed 2013 Aug 16. 10.1093/jmedent/20.5.568 6644754

[27] Jones TM, Balmford A, Quinnell RJ (2000) Adaptive female choice for middle-aged mates in a lekking sandfy. Proc R Soc Lond B 267: 681–686. Available: http://www.ncbi.nlm.nih.gov/pubmed/10821613. Accessed 2013 Aug 16. 10.1098/rspb.2000.1056 PMC1690592 10821613

[28] Ottoni EB (2000) EthoLog 2.2: a tool for the transcription and timing of behavior observation sessions. Behav Res Meth Ins C 32: 446–449. Available: http://www.ncbi.nlm.nih.gov/pubmed/11029818. Accessed 2013 Aug 16. 10.3758/bf03200814 11029818

[29] Lazareva AA, Roman G, Mattox W, Hardin PE, Dauwalder B (2007) A role for the adult fat body in *Drosophila* male courtship behavior. PLoS Genet 3: e16. Available: http://www.pubmedcentral.nih.gov/articlerender.fcgi?artid=1781494&tool=pmcentrez&rendertype=abstract. Accessed 2013 Aug 16. 10.1371/journal.pgen.0030016 PMC1781494 17257054

[30] Jones TM, Quinnell RJ, Balmford A (1998) Fisherian flies: Benefits of female choice in a lekking sandfly. Proc R Soc Lond 265: 1651–1657. Available: http://www.jstor.org/discover/10.2307/51137?uid=24156&uid=3737664&uid=5909624&uid=2&uid=3&uid=67&uid=24155&uid=62&sid=21101378063307. Accessed 2013 Aug 16.

[31] Ilango K (2005) Structure and function of the spermathecal complex in the phlebotomine sandfly *Phlebotomus papatasi* Scopoli (Diptera: Psychodidae): I. ultrastructure and histology. J Bioscience 30: 711–731. Available: http://www.ncbi.nlm.nih.gov/pubmed/16388145. Accessed 2013 Aug 16. 10.1007/BF02703571 16388145

[32] Dallai R, Baccetti B, Mazzini M (1984) The spermatozoon of three species of *Phlebotomus* (Phlebotominae) and the acrosomal evolution in nematoceran dipterans. Int J Insect Morphol & Embryol 13: 1–10.

[33] Forattini OP (1973) Entomologia Médica. Vol. 4, Psychodidae, Phlebotominae, Leishmanioses, Bartonelose. São Paulo: Edgar Blücher & Editora da Universidade de São Paulo. 658 p.

[34] Riemann JG (1973) Ultrastructure of the ejaculatory duct region producing the male housefly accessory material. J Insect Physiol 19: 213–223. Available: http://www.sciencedirect.com/science/article/pii/0022191073902345. Accessed 2013 Aug 16. 10.1016/0022-1910(73)90234-5 4688689

[35] Meikle DB, Sheehan KB, Phillis DM, Richmond RC (1990) Localization and longevity of seminal-fluid esterase 6 in mated female *Drosophila melanogaster* J Insect Physiol 36: 93–101. Available: http://linkinghub.elsevier.com/retrieve/pii/002219109090179J. Accessed 2013 Aug 16.

[36] Lee JJ, Klowden MJ (1999) A male accessory gland protein that modulates female mosquito (Diptera: Culicidae) host-seeking behavior. J Am Mosquito Control 15: 4–7. Available: http://www.ncbi.nlm.nih.gov/pubmed/10342262. Accessed 2013 Aug 16. 10342262

[37] Bray DP, Hamilton JGC (2007) Courtship behaviour in the sandfly *Lutzomyia longipalpis*, the New World vector of visceral leishmaniasis. Med Vet Entomol 21: 332–338. Available: http://www.ncbi.nlm.nih.gov/pubmed/18092971. Accessed 2013 Aug 16. 10.1111/j.1365-2915.2007.00700.x 18092971

[38] Souza NA, Andrade-Coelho CA, Vigoder FM, Ward RD, Peixoto AA (2008) Reproductive isolation between sympatric and allopatric Brazilian populations of *Lutzomyia longipalpis* s.l. (Diptera: Psychodidae). Mem Inst Oswaldo Cruz 103: 2016–2019. Available: http://www.scielo.br/scielo.php?script=sci_arttext&pid=S0074-02762008000200017. Accessed 2013 Aug 16. 10.1590/s0074-02762008000200017 18425278

[39] Jones M (2001) A Potential Cost of Monandry in the Lekking. J Insect Behavior 14: 385–399. Available: http://link.springer.com/article/10.1023%2FA%3A1011127514317#page-1. Accessed 2013 Aug 16.

[40] Vitta ACR, Lorenzo MG (2009) Copulation and mate guarding behavior in Triatoma brasiliensis (Hemiptera: Reduviidae). J Med Entomol 46: 789–795. Available: http://www.ncbi.nlm.nih.gov/pubmed/19645281. Accessed 2013 Aug 16. 10.1603/033.046.0409 19645281

[41] Nattero J, Leonhard G, Rodríguez CS, Crocco L (2011) Influence of the quality and quantity of blood ingested on reproductive parameters and life-span in *Triatoma infestans* (Klug). Acta tropica 119: 183–187. Available: http://www.ncbi.nlm.nih.gov/pubmed/21672510. Accessed 2013 Aug 16. 10.1016/j.actatropica.2011.05.015 21672510

[42] Yuval B (2006) Mating systems of blood-feeding flies. Ann Rev Entomol 51: 413–440. Available: http://www.ncbi.nlm.nih.gov/pubmed/16332218. 2013. Accessed 2013 Aug 16. 10.1146/annurev.ento.51.110104.151058 16332218

[43] Souza NA, Andrade-Coelho CA, Silva VC, Ward RD, Peixoto AA (2009) Life cycle differences among Brazilian sandflies of the *Lutzomyia longipalpis* sibling species complex. Med Vet Entomol 23: 287–292. Available: http://www.ncbi.nlm.nih.gov/pubmed/19712160. Accessed 2013 Aug 16. 10.1111/j.1365-2915.2009.00818.x 19712160

[44] Valenta D, Killick-Kendrick R, Killick-Kendrick M (2000) Courtship and mating by the sandfly *Phlebotomus duboscqi*, a vector of zoonotic cutaneous leishmaniasis in the Afrotropical region. Med Vet Entomol 14: 207–212. Available: http://www.ncbi.nlm.nih.gov/pubmed/10872866. Accessed 2013 Aug 16. 10.1046/j.1365-2915.2000.00225.x 10872866

[45] Bray DP, Hamilton JGC (2007) Courtship behaviour in the sandfly *Lutzomyia longipalpis*, the New World vector of visceral leishmaniasis. Medical and veterinary entomology 21: 332–338. Available: http://www.ncbi.nlm.nih.gov/pubmed/18092971. Accessed 2013 Aug 16. 10.1111/j.1365-2915.2007.00700.x 18092971

[46] Bretman A, Westmancoat JD, Chapman T (2013) Male control of mating duration following exposure to rivals in fruitflies. J Insect Physiol 59: 824–827. Available: http://www.ncbi.nlm.nih.gov/pubmed/23727302. Accessed 2013 Aug 16. 10.1016/j.jinsphys.2013.05.011 PMC3885974 23727302

