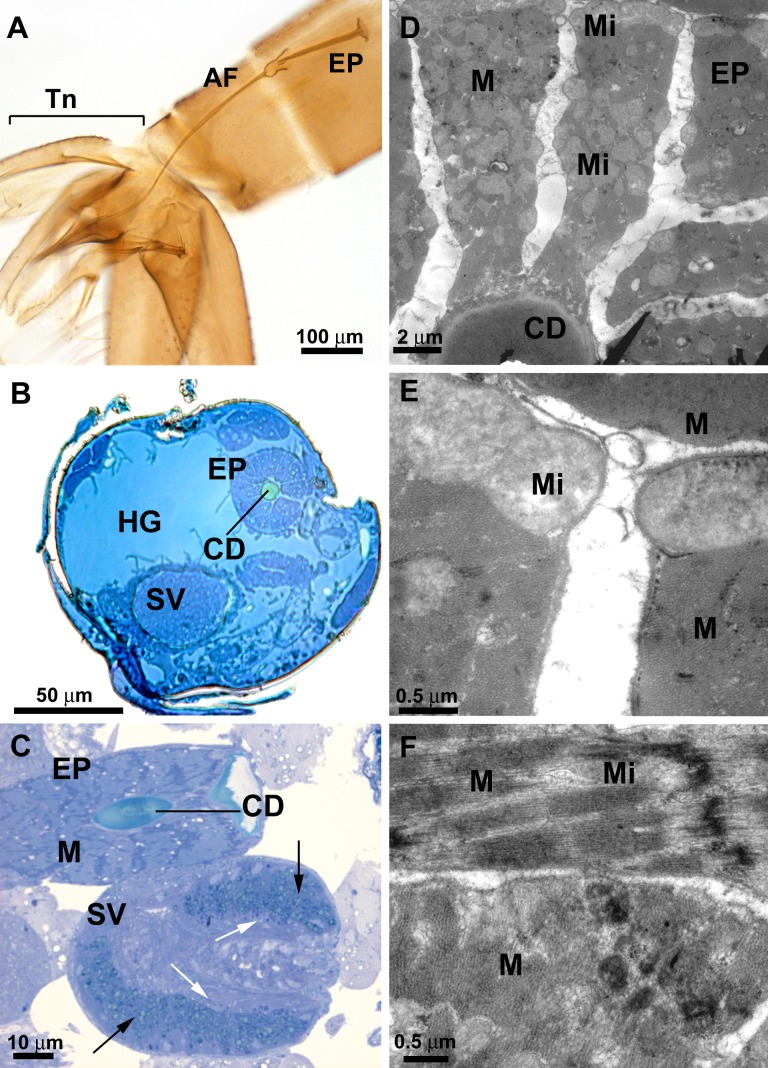# Correction: Fine Structure of the Male Reproductive System and Reproductive Behavior of *Lutzomyia longipalpis* Sandflies (Diptera: Psychodidae: Phlebotominae)

**DOI:** 10.1371/annotation/7d6a463d-41b1-4225-b09b-edecc565071c

**Published:** 2014-01-02

**Authors:** Carolina N. Spiegel, Jorge A. C. Bretas, Alexandre A. Peixoto, Felipe M. Vigoder, Rafaela V. Bruno, Maurilio J. Soares

There is an error in Figure 6A. The genitalia in Figure 6A belongs to another species (Evandromyia lenti), and not to Lutzomyia longipalpis as described in the legend. Please see the corrected Figure 6A of Lutzomyia longipalpis here: 

**Figure:**