# Supplementary figures and images for: Fine Structure of the Male Reproductive System and Reproductive Behavior of Lutzomyia longipalpis Sandflies (Diptera: Psychodidae: Phlebotominae)
Source: PLoS One. 2013 Sep 13;8(9):e74898. doi: 10.1371/journal.pone.0074898 (PMC3772895; doi:10.1371/journal.pone.0074898)

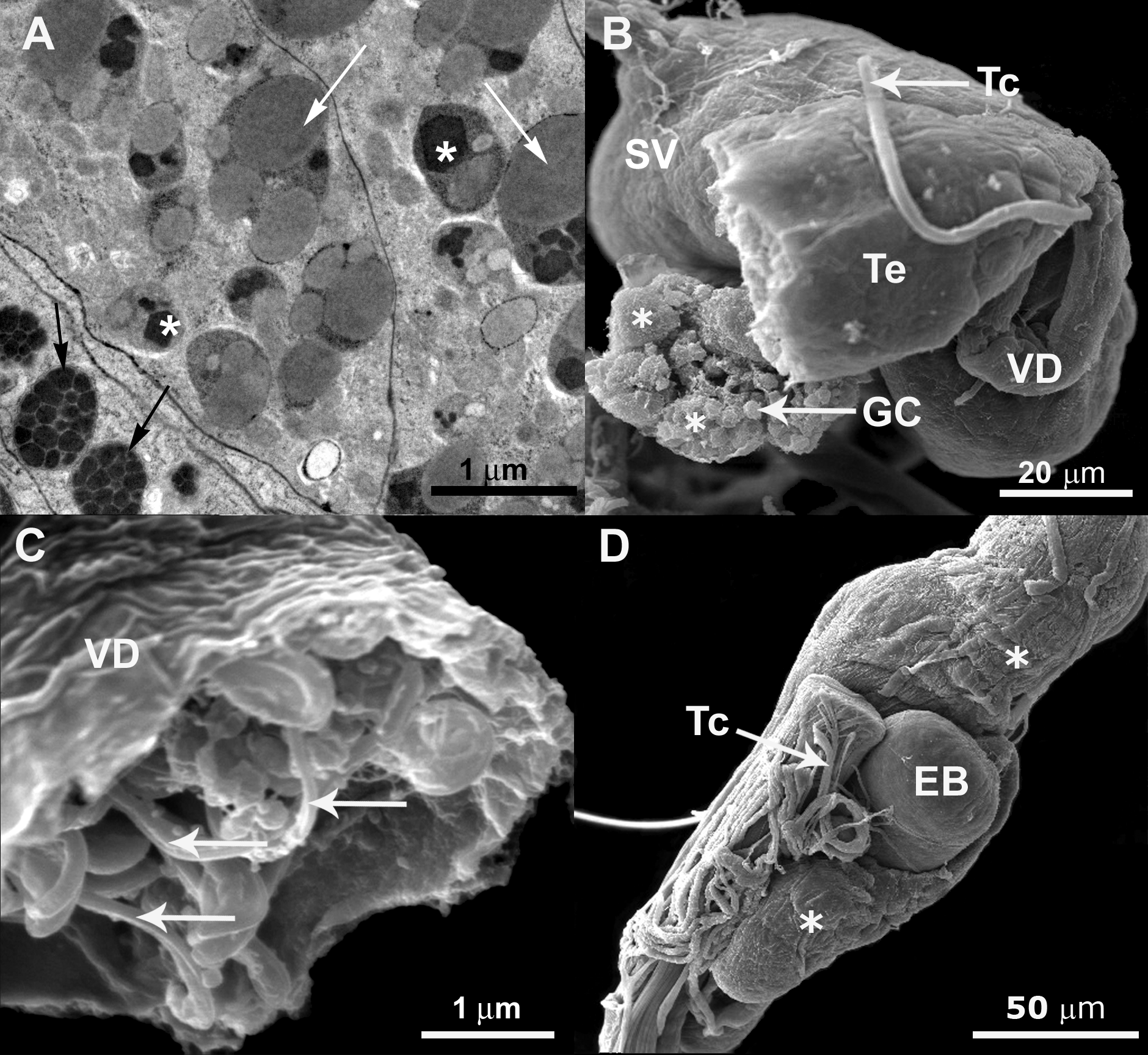

Supplement: Figure S1 — L. longipalpis male reproductive system. A) TEM detail of cytoplasmic granules presenting a crystalloid inclusion (asterisk) in cells at the peripheral epithelium of the seminal vesicle wall. Note the difference in density between granules in cells from the outer epithelial wall (white arrows) and in cells from the invaginated epithelium of the seminal vesicle wall (black arrows). B) SEM section of a testis (Te), with groups of germ cells (gc) in primary stage of development. Note the cyst (asterisks) formed by germ cells and cystocytes. The seminal vesicle (sv), a vas deferens (vd) and trachea (tc) can be also observed. C) SEM section of a vas deferens (vd) from an insect six hours after emergence. Note the presence of flagellate spermatozoa (arrows). D) SEM detail of the ejaculatory duct, formed by a central bulb, and the ejaculatory bulb (eb) surrounded by compressor muscles (asterisks) associated with several tracheas (tc). (TIF) [file pone.0074898.s001.tif]
